# Viloxazine occupies the 5-HT_2C_ receptor in the *Macaca fascicularis* brain in vivo: a [^11^C]CIMBI-36 positron emission tomography study

**DOI:** 10.1093/ijnp/pyag034

**Published:** 2026-06-23

**Authors:** Jennie Garcia-Olivares, Brittney Yegla, Khanum Ridler, Gaia Rizzo, Zhen Fan, Amy Llopis Amenta, Anton Forsberg Moren, Christer Halldin, Vladimir Stepanov, Jennifer Koch, Chungping Yu, Jonathan Rubin, Vladimir Maletic, Eugenii A Rabiner

**Affiliations:** Supernus Pharmaceuticals, Inc., Rockville, MD, United States; Supernus Pharmaceuticals, Inc., Rockville, MD, United States; Perceptive Discovery, London, United Kingdom; Perceptive Discovery, London, United Kingdom; Perceptive Discovery, London, United Kingdom; Perceptive Discovery, London, United Kingdom; Karolinska Institutet, Stockholm, Sweden; Karolinska Institutet, Stockholm, Sweden; Karolinska Institutet, Stockholm, Sweden; Supernus Pharmaceuticals, Inc., Rockville, MD, United States; Supernus Pharmaceuticals, Inc., Rockville, MD, United States; Supernus Pharmaceuticals, Inc., Rockville, MD, United States; University of South Carolina School of Medicine, Greenville, SC, United States; Duke University, Durham, NC, United States; Perceptive Discovery, London, United Kingdom

**Keywords:** viloxazine, PET, 5-HT_2C_, [^11^C]CIMBI-36, ADHD

## Abstract

**Objective:**

Efficacy of viloxazine ER (extended-release capsules; Qelbree®) for attention-deficit/hyperactivity disorder (ADHD) is attributed to its ability to increase extracellular norepinephrine and dopamine in the medial prefrontal cortex by inhibiting norepinephrine transporters (NET); however, studies also suggest potentially relevant activity at serotonin (5-HT) receptors. In this study, we evaluated the target engagement by viloxazine at 5-HT_2_ receptor subtypes, in species with close similarities with humans, and whether viloxazine engages 5-HT_2_ receptor subtypes within a clinically relevant plasma concentration range.

**Methods:**

This study utilized positron emission tomography (PET) and was conducted to measure the relationship between viloxazine plasma concentration and the changes in binding of the agonist radioligand [^11^C]CIMBI-36 to 5-HT_2C_/5-HT_2A_ receptors in the brain of *Macaca fascicularis* monkeys.

**Results:**

Viloxazine administration reduced the binding of [^11^C]CIMBI-36 at 5-HT_2C_ receptors at a concentration 10- to 20-fold lower than at 5-HT_2A_ receptors (EC_50_: 4.1 vs 45.1 μM, respectively), consistent with receptor binding affinities previously measured in vitro (K_i_: 0.66 vs 16.0 μM, respectively). Unbound viloxazine plasma concentrations during these PET scans were compared to human unbound concentrations at doses used to treat ADHD. Our data suggest that at clinically relevant plasma concentrations, viloxazine occupies 60%-72% of 5-HT_2C_ receptors.

**Conclusion:**

Our results demonstrate that at unbound viloxazine plasma concentrations comparable with those clinically relevant for the treatment of ADHD, viloxazine occupies a high proportion of 5-HT_2C_ receptors. Occupancy of and potential functional activity at 5-HT_2C_ receptors could therefore contribute to the therapeutic activity of viloxazine in addition to inhibition of NET.

## Graphical Abstract

**Graphical Abstract:**
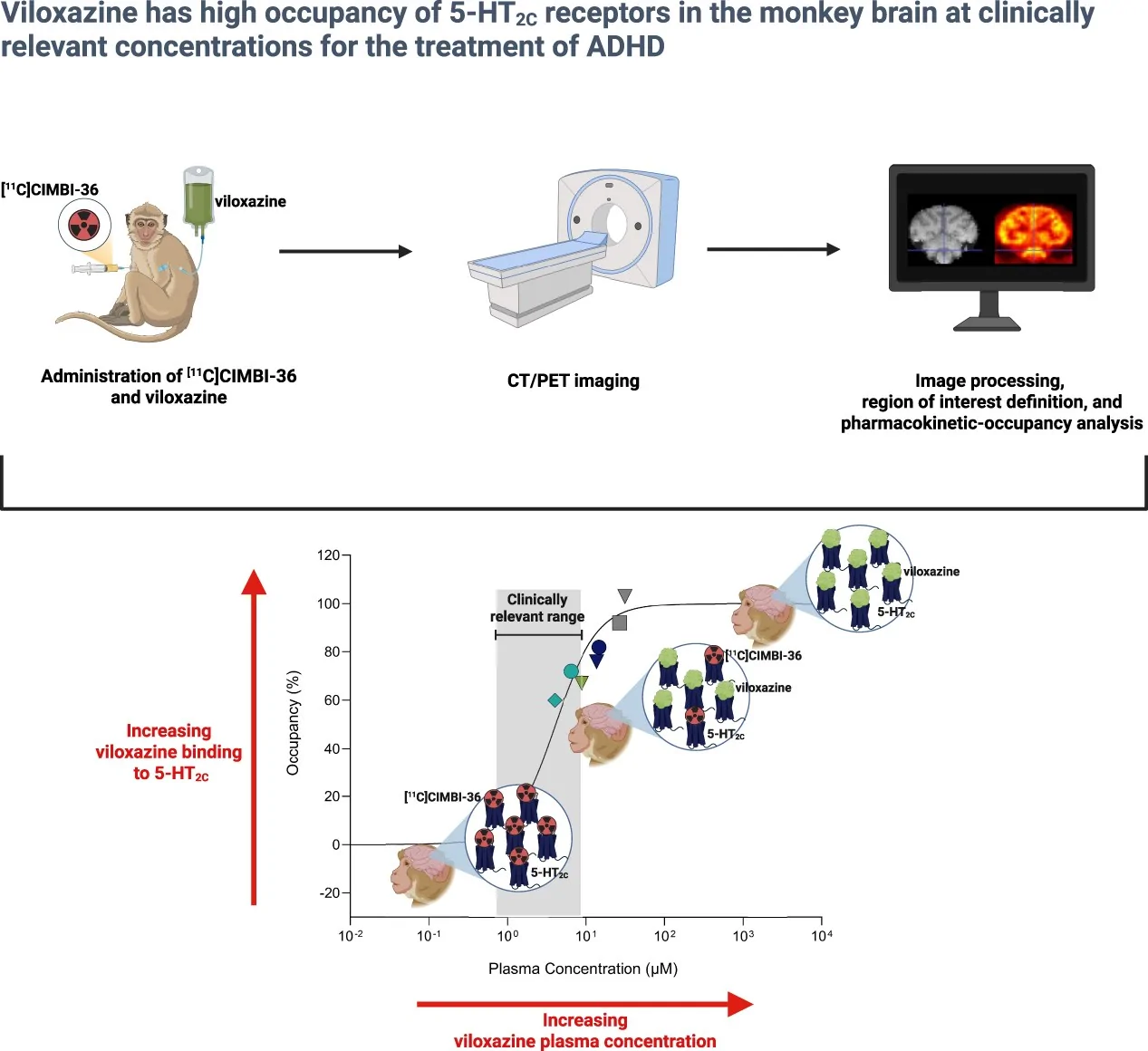

**Significant outcomes**

- This PET imaging study demonstrates that viloxazine, a medication FDA-approved for the treatment of ADHD, blocks the binding of a 5-HT_2_ radioligand agonist ([^11^C]CIMBI-36) in the choroid plexus of the cynomolgus monkey brain, consistent with occupancy of the 5-HT_2C_ receptors. Based on these results, high levels of occupancy of 5-HT_2C_ receptor occupancy are expected at unbound viloxazine plasma concentrations comparable with those clinically relevant for the treatment of ADHD.

**Limitations**

- [^11^C]CIMBI-36 binds 5-HT_2C_ receptors in brain regions that may differ from those responsible for viloxazine’s clinically relevant effects in ADHD, limiting the study’s relevance to its ADHD mechanism.
- The limited number of animals and doses may contribute to data variability.
- Clinically relevant viloxazine plasma concentrations were estimated assuming similar drug distribution in non-human primates and humans and direct plasma-brain diffusion.
- Only one baseline scan was performed per animal, so baseline variability may have influenced the estimates.
- The study did not determine whether viloxazine or endogenous serotonin displaced [^11^C]CIMBI-36 at 5-HT_2A/2C_ receptors, leaving the mechanism at these receptors unclear.

## Introduction

An extended-release (ER) formulation of viloxazine is approved for use in the United States as a once-daily treatment for attention-deficit/hyperactivity disorder (ADHD). Previously studied and available in Europe as an antidepressant, viloxazine was designated a selective norepinephrine reuptake inhibitor (NRI) because it showed moderate affinity for the norepinephrine (NE) transporter (NET) while displaying negligible activity at serotonin (5-HT) and dopamine (DA) transporters (SERT and DAT, respectively).^1^^,^^2^ However, this early classification paints an incomplete picture of viloxazine’s pharmacologic activity, first by disregarding the ability of NET inhibitors to also increase dopamine (NETs can transport DA as well as NE in brain areas such as the prefrontal cortex [PFC] where DATs are scarce),^3^^,^^4^ and second by ignoring a body of work conducted in the 1970s and 1990s, showing that viloxazine also modulates 5-HT transmission.^1^^,^^5–8^ The ability to augment DA neurotransmission is of high relevance for ADHD treatment, and an emerging body of research suggests modulation of 5-HT receptors may also be relevant to its mechanism in the treatment of ADHD.^9^ Therefore, Supernus Pharmaceuticals, Inc. (the US sponsor of viloxazine ER) conducted a series of experiments to more accurately characterize the pharmacologic effects of viloxazine, in order to better understand which effects may be therapeutically relevant to its mechanism of action in ADHD.^10–12^

An initial series of in vitro experiments, confirmed early studies and showed viloxazine selectively inhibits NET (without substantial inhibition of SERT) and has binding at several serotonin receptors including 5-HT_2C_ (K_i_: 0.66 μM)_,_ 5-HT_2B_ (K_i_: 0.83 μM), and 5-HT_7_ (K_i_:1.9 μM), with weaker binding to 5-HT_2A_ (K_i_:16.3 μM)_._^10^^,^^13^ Specifically, viloxazine showed agonist effects at 5-HT_2C_ (EC_50_: 1.6 μM [Ca^2+^ assay]) and antagonist effects at 5-HT_2B_ (IC_50_: 27 μM [IP_1_ assay]) and 5-HT_7_ (IC_50_: 6.7 μM [cAMP assay]), with weak antagonistic effects at 5-HT_2A_ (IC_50_: >300 μM [IP_1_ assay]).^10^^,^^14^ These in vitro findings need to be bolstered by in vivo studies, but if viloxazine binds to 5-HT_2C_, 5-HT_2B_, and/or 5-HT_7_ in vivo, it is possible this action may play a role in viloxazine’s therapeutic activity.

To evaluate whether viloxazine could bind 5-HT_2_ receptors in vivo, we performed a positron emission tomography (PET) imaging study in non-human primates (NHPs), *Macaca fascicularis*, using [^11^C]CIMBI-36. [^11^C]CIMBI-36 was chosen as it is brain permeable radioligand agonist and has high affinity for 5-HT_2A_ and 5-HT_2C_ receptors (K_i_ [nM]: 5-HT_2A_ = 0.5-0.8; 5-HT_2C_ = 1.7) and high selectivity over other targets in vitro (>120-fold).^15^ Further, occupancy of [^11^C]CIMBI-36 at either 5-HT_2A_ and/or 5-HT_2C_ receptors can be differentiated as previous studies demonstrated that binding of [^11^C]CIMBI-36 in cortical regions is almost entirely attributable to 5-HT_2A_ receptor binding, while its binding in the choroid plexus is almost entirely attributable to 5-HT_2C_ receptor binding.^16^^,^^17^ Therefore, we aimed to determine whether viloxazine could block the binding of [^11^C]CIMBI-36 at 5-HT_2C_ and 5-HT_2A_ receptors, and to evaluate the relationship between the plasma concentration of viloxazine and the levels of target occupancy at these receptors. Based on our prior in vitro experiments^10^^,^^12^ showing potent binding affinity and agonism (low EC_50_ values) of viloxazine for 5-HT_2C_, and weak binding affinity and antagonism (high IC_50_ values) of viloxazine towards 5-HT_2A,_ we hypothesized that in vivo viloxazine would have high occupancy at 5-HT_2C_ and low occupancy at 5-HT_2A_ receptors, and that 5-HT occupancy would occur within clinically relevant plasma levels for the treatment of ADHD.

## Materials and methods

### Animals

The study was approved by the Animal Ethics Committee of the Swedish Animal Welfare Agency (Dnr 10367-2019) and was performed according to standard operating procedures including “Guidelines for planning, conducting and documenting experimental research” (Dnr 4820/06-600) at the Karolinska Institutet. All procedures were performed in accordance with the United Kingdom Animals (Scientific Procedures) Act 1986 and European Union Directive 2010/63/EU.

Four NHPs (*Macaca fascicularis*) were included in the study (see Supplemental Table S1 for individual animal details). All subjects were housed at the Astrid Fagræus Laboratory of the Karolinska Institutet (Solna, Sweden). For details on NHP care see Supplemental Methods.

### Test and control articles

Unlabeled N-(2[^11^C]methoxybenzyl)-2,5-dimethoxy-4-bromophenethylamine ([^11^C]CIMBI-36) and its precursor (tert-butyl 4-bromo-2,5-dimethoxyphenethyl (2-hydroxybenzyl)carbamate, CIMBI-37, were produced by the University of Copenhagen’s Department of Drug Design and Pharmacology (Copenhagen, Denmark). [^11^C]CIMBI-36 was prepared at the Karolinska Institutet (Stolkholm, Sweden) as described elsewhere^16^^,^^17^ with minor modifications. For description of preparation see Supplemental Methods.

Viloxazine hydrochloride, lot 1000005854 (Bachem SA), was supplied by Supernus Pharmaceuticals, Inc. (Rockville, MD). For description of preparation see Supplemental Methods.

### Study design

**Figure 1:**
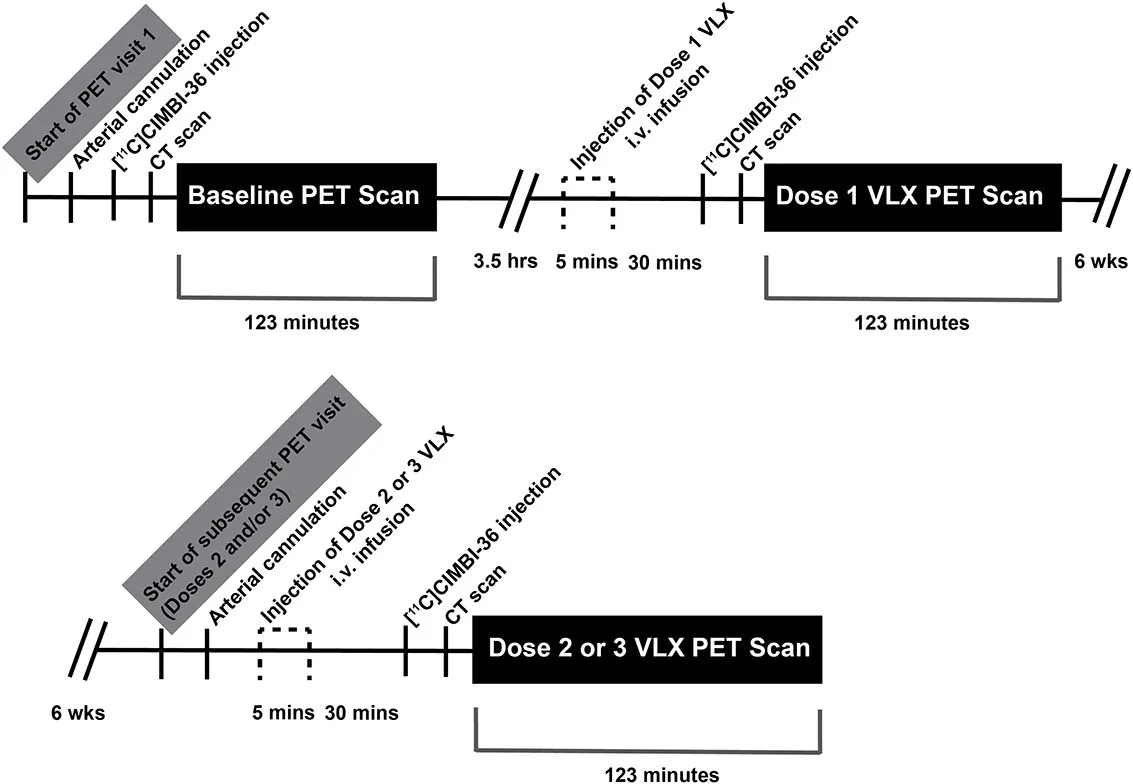
Study design for baseline, first dose viloxazine, and subsequent VLX PET scans. Abbreviations: PET = positron emission tomography; VLX = viloxazine.

A total of 14 PET scans were performed in 4 NHPs (ID: 001, 002, 003, and 004) on 9 experimental days. Experimental procedures are described in detail below but briefly began with performing a head computed tomography (CT) scan before each PET scan in order to perform attenuation correction of [^11^C]CIMBI-36 scans. Before any administration of viloxazine, each NHP had a baseline PET scan conducted to determine normal [^11^C]CIMBI-36 distribution (Figure 1). Each baseline scan consisted of arterial cannulation, then injection of [^11^C]CIMBI-36 (dose: 162 ± 28 MBq, molar activity 376.5 ± 291.7 GBq/μmol, injected mass: 0.27 ± 0.18 μg) followed by 123 min of data acquisition. At 3.5 h after the baseline scan, an IV infusion of viloxazine was administered over 5 min, followed 30 min later by a second [^11^C]CIMBI-36 injection and a second PET scan. Due to the number of blood samples taken prior to and throughout the PET scan procedure, a minimal rest period of 6 weeks was required after each scan. Therefore, subsequent scans to test additional doses of viloxazine were conducted at least 6-weeks after the initial scanning date. The procedure on subsequent scanning dates began with the administration of viloxazine, as portrayed in Figure 1. The baseline scan was not repeated for subsequent viloxazine doses, all subsequent doses were compared to the original baseline scan.

Subject NHP002 was withdrawn from the study, after successfully completing the baseline and a post-baseline scan, due to an issue unrelated to the test articles, and was replaced by another subject (NHP004). While preparing NHP002 for a second post-baseline PET scan, the subject experienced respiratory difficulties after a problematic intubation, which required that the subject be euthanized; the subject was not exposed to viloxazine during this occasion.

Initial dose selection in the experiment was guided by prior studies in baboons, in which 12 mg/kg of viloxazine was the highest administered dose that did not result in prohibitive adverse events.^18^ Based on results using the 12 mg/kg dose, subsequent doses were selected in an adaptive design in order to optimally describe the relationship between the plasma concentration of viloxazine and occupancy of the 5-HT_2_ receptors over a therapeutic concentration range based on unbound plasma concentrations obtained from pediatric and adult population pharmacokinetic models (*C*_max_ 0.8-7.2 μM), that were developed using steady-state plasma concentrations from over 500 participants in phase 3, ADHD clinical trials (Supernus data on file).

### Blood sampling for determination of [^11^C]CIMBI-36 radioactivity and radiometabolite analysis

For each PET scan, the fraction of [^11^C]CIMBI-36 plasma radioactivity was determined from the unchanged parent radioligand in arterial whole blood samples as described previously.^17^^,^^19^ See Supplemental Methods for details.

### Determination of viloxazine plasma concentrations

Venous blood samples for viloxazine plasma concentrations were collected 1-min pre- (−1 min), and 15, 30, 60, and 120 min post-[^11^C]CIMBI-36 administration. Viloxazine plasma concentrations were determined at Lablytica Life Science (Uppsala, Sweden), using ultra performance liquid chromatography followed by tandem mass spectrometry (MS/MS) detection (lower limit of quantification = 1.5 ng/mL). See Supplemental Methods for details.

### PET acquisition

Anesthesia was induced by intramuscular injection of ketamine hydrochloride (approximately 10 mg/kg) at Astrid Fagræus Laboratory and maintained by the administration of a mixture of sevoflurane (2.0%-8.0%), oxygen and medical air with endotracheal intubation. Body temperature and fluid balance were maintained and heart rate, blood pressure, respiratory rate, and oxygen saturation were monitored continuously.

PET/CT scans were conducted as previously described^17^ using a MultiScan™ Large Field of view Extreme Resolution (LFER150) PET/CT (Mediso; Budapest, Hungary). Dynamic emission PET images were acquired for 123 min per scan per procedure as described in Finnema et al.^16^ and reconstructed in 34 frames (20 s × 9, 1 min × 3, 3 min × 5, and 6 min × 17) using the 3D iterative reconstruction algorithm (Tera-Tomo™), with 5 iterations and 9 subsets including modeling of the point spread function. Corrections were applied for attenuation, scatter and positron range, dead time, and decay correction.

### PET image processing and region-of-interest definition

The Cognitive Neuroscience Centre-National Centre for Scientific Research [France] neuroanatomical atlas^20^ was non-linearly wrapped to the subject’s structural magnetic resonance image (acquired prior to start of study) to enable automated pre-defined regions of interest (ROIs). The ROI time-activity data were sampled from ROIs determined a priori including: the frontal cortex (high 5-HT_2A_ [^11^C]CIMBI-36 binding), choroid plexus (high 5-HT_2C_ [^11^C]CIMBI-36 binding), and hippocampus (mixed 5-HT_2A_ and 5-HT_2c_ [^11^C]CIMBI-36 binding). The cerebellum was used as a reference region to estimate [^11^C]CIMBI-36 non-displaceable binding.

The hippocampus and choroid plexus were manually delineated using PET and MRI images (software used: ITKSnap®). The region for the choroid plexus was defined around the radioactivity in the fourth ventricle using fused PET and MRI images, as described previously.^16^ The hippocampus was defined around the radioactivity in the head and tail of the hippocampus, since the automatic atlas segmentation was deemed too variable and generous in the region definition.

ROIs defined on the MRI images were applied to dynamic PET data after registration of the MRI to the PET image to generate time-activity curves (TACs), with activity concentrations expressed as standardized uptake values, calculated as the ratio of radioactivity in a ROI to the injected dose of radioactivity per kg of body weight:



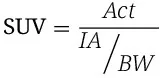



where *Act* is the measured radioactivity concentration (kBq/mL), *IA* is the injected radioactivity (MBq) and *BW* is the subject’s body weight (kg).

### Arterial input function measurement and arterial blood data processing

Continuous blood data were scaled (ie, calibrated) to match overlapping discrete whole blood samples, and then merged with the remaining discrete whole blood data to form a whole blood activity curve covering the full length of the scan. Activity measurements from the discrete plasma samples were divided by the corresponding whole blood data to form plasma-over-blood (POB) data. The POB data were fitted to an exponential approaching to a constant model in order to interpolate the relationship. The POB model fit curve was multiplied by the whole blood curve to give an estimated total plasma curve (Supplemental Figures S1-S4).

The [^11^C]CIMBI-36 plasma parent fraction (metabolite) data were fitted to a sigmoid model. The resulting fitted parent fraction profile was multiplied by the total plasma curve and then smoothed post-peak using a multi-exponential fit to give the required parent plasma input function. For each scan, a time delay was fitted and applied to the input function to account for any delay between blood sampling measurements and tissue data from the PET tomograph. The resulting parent plasma input function was used as input for the kinetic modeling processes described below.

### Kinetic modeling

Regional total volume of distribution (*V_T_*) was derived from kinetic analysis using the multilinear analysis 1 (MA1) with t^*^ = 30 min (chosen based on previous experience as the optimal point for the application of the MA1 model to [^11^C]CIMBI-36 data) as previously described.^17^^,^^21^^,^^22^ In past experience, the MA1 model provided very similar *V_T_* estimates to the 2-tissue compartmental model but with greater stability and significantly lower incidence of non-convergence for regional *V_T_* data. Regional *V_T_* values are shown in Supplemental Table S2. Regional non-displaceable binding potential (BP_ND_) relative to the cerebellum was calculated as:^23^


$${\mathrm{BP}}_{\mathrm{ND}}^{\mathrm{ROI}}=\frac{V_T^{\mathrm{ROI}}}{V_T^{\mathrm{cerebellum}}}-1$$

### Estimation of and the relationship between Pharmacokinetic (PK)-BP_ND_ parameters

The percentage reduction in binding potential (change in BP_ND_) in an ROI after viloxazine treatment was quantified as:


$$\Delta{\mathrm{BP}}_{\mathrm{ND}}=100 \times \left(1-\frac{{\mathrm{BP}}_{\mathrm{ND}}^{\mathrm{post}-\mathrm{dose}}}{{\mathrm{BP}}_{\mathrm{ND}}^{\mathrm{baseline}.}}\right).$$

In order to characterize the relationship between unbound viloxazine plasma concentration and *Δ*BP_ND_ (or % of occupancy), *Δ*BP_ND_ was plotted against corresponding measured plasma concentration data and a maximum effect (*E*_max_) model was fitted to derive a half-maximal effective concentration (EC_50_) (viloxazine plasma concentration corresponding to 50% receptor occupancy at the time point studied):


$$\Delta{\mathrm{BP}}_{\mathrm{ND}}=100\times \frac{C_p}{C_p+{\mathrm{EC}}_{50}},$$

where *Cp* is the plasma concentration of viloxazine (as determined above and detailed in the Supplemental Methods) measured at a representative time point of 15 min post [^11^C]CIMBI-36 injection (40 min post-viloxazine infusion).

## Results

### [^11^C]CIMBI-36 PET imaging and pharmacokinetic analyses

**Figure 2:**
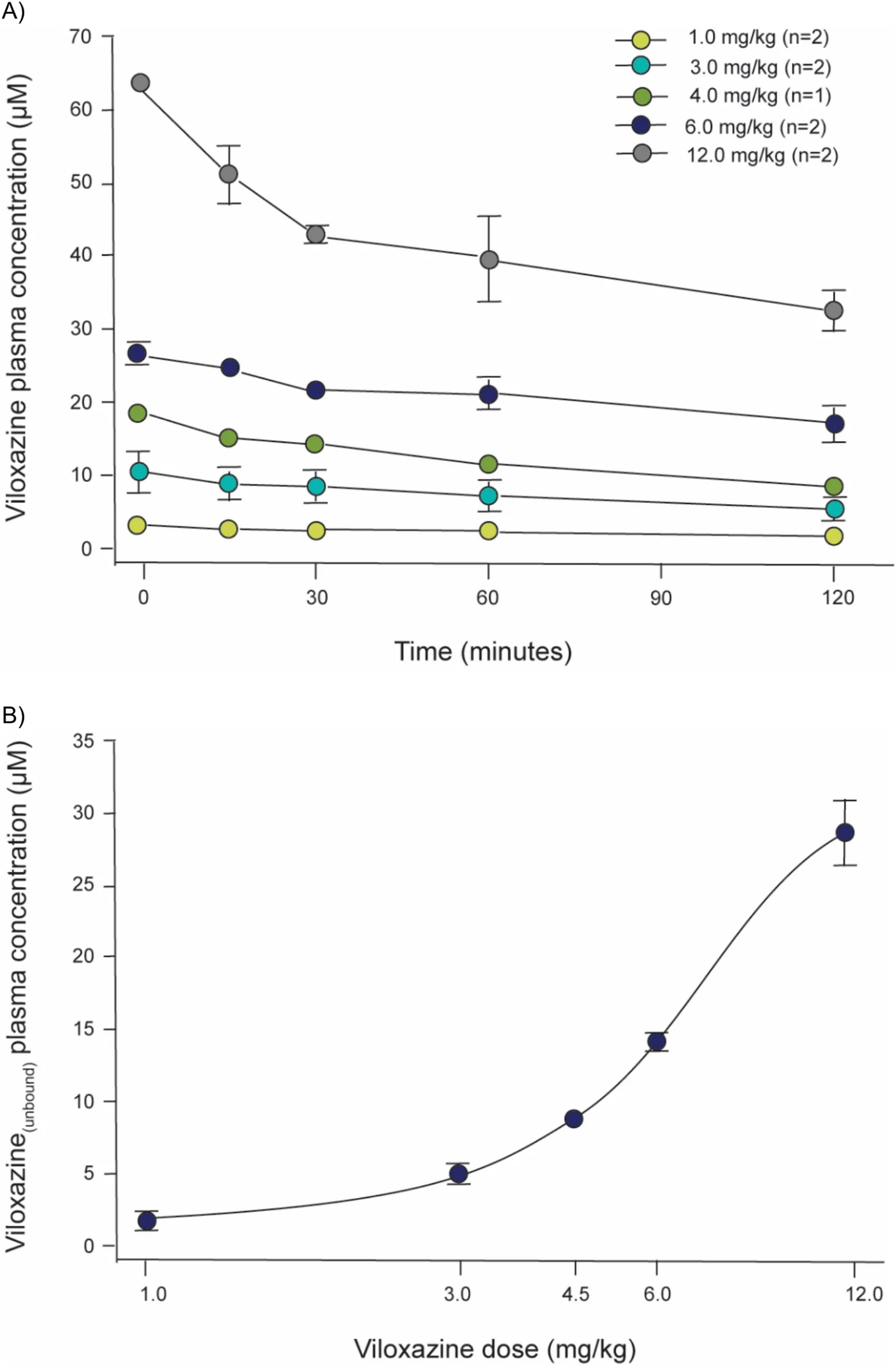
Viloxazine plasma pharmacokinetics. Venous blood was drawn during PET scan procedure after intravenous (i.v.) bolus infusion of viloxazine (5-min infusion time at *t* = −30) at *t* = −1, 15, 30, 60 and 120 min. (A) Time course of total viloxazine concentration in plasma (μM). (B) Pharmacokinetic analysis of blood samples. Dose on x-axis is logarithmic. The fraction of unbound viloxazine was estimated after normalization by 0.57 (measured fraction of unbound viloxazine in cynomolgus monkey). Symbols represent mean ± SEM, *n* is the number of samples/dosing group. Of note, the predose (−1 min) venous blood sample was missed for the initial blocking scan in NHP001 at the viloxazine 12 mg/kg dose. Abbreviation: PET = positron emission tomography.

**Figure 3:**
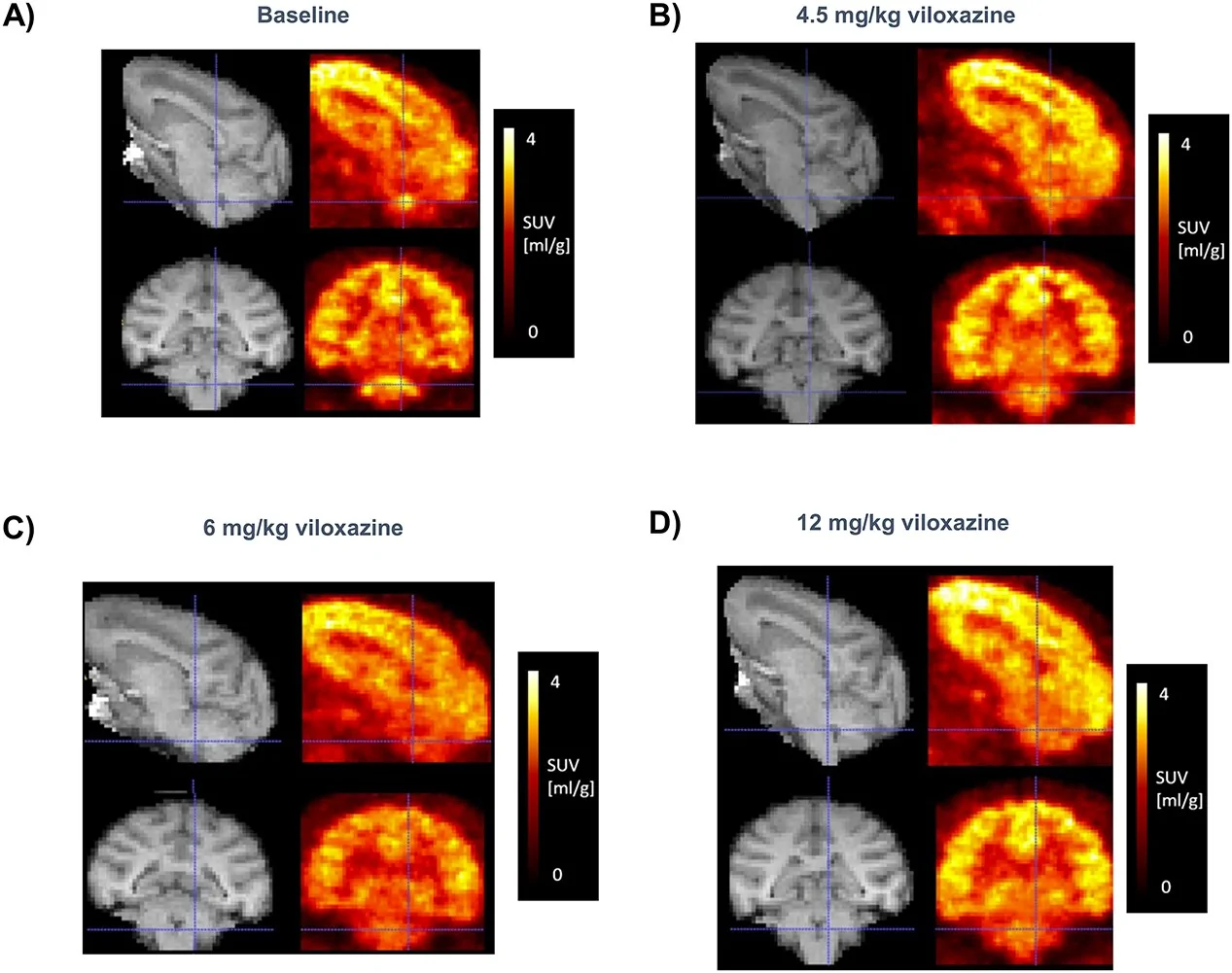
Representative [^11^C]CIMBI-36 CT and PET images of NHP001 showing expected radioligand uptake at (A) baseline, and after administration of (B) 4.5, (C) 6, and (D) 12 mg/kg of viloxazine scaled to SUVs along with an MRI template of the brain. Abbreviations: CT = computed tomography; PET = positron emission tomography; SUV = standardized uptake value.

In baseline scans, [^11^C]CIMBI-36 demonstrated regional binding consistent with previous experiments and the known distribution of 5-HT_2A_ and 5-HT_2C_ receptors in the brain. PET data and viloxazine pharmacokinetics from the initial 12 mg/kg dose scans in 2 NHPs were used to select subsequent doses of viloxazine with the aim of adequately characterizing the dose occupancy curves for the 5-HT_2A_ and 5-HT_2C_ receptors. The 12 mg/kg dose of viloxazine resulted in high total unbound plasma concentrations (Figure 2a) and was able to lower the signal of [^11^C]CIMBI-36, consistent with viloxazine binding to the 5-HT_2A_ and/or 5-HT_2C_ receptor(s) (representative scan for NHP001 in Figure 3). Following the results of the 12 mg/kg dose, subsequent scans were conducted using 4 additional doses of viloxazine (1.0, 3.0, 4.5, and 6.0 mg/kg) distributed among 4 NHPs (scans shown in Supplemental Figures S5-S7). A total of 13 scans were performed, including baseline scans and subsequent scans with viloxazine treatment. See Supplemental Table S3 for record of each NHP scan and order of dose testing. Attempts were made to study each dose in 2 different NHPs (the 4.5 mg/kg dose was only tested once). Non-human primates were chosen for each scan based on the elapsed time since their previous scan. Viloxazine plasma concentrations were dose dependent (Figure 2b) and remained fairly stable over time during the 123-min scan period (Figure 2a). Kinetic modeling with MA1 produced acceptable model fits to tissue TAC data in all cases (representative TACs and MA1 model fits in Supplemental Figures S8-S11).

### Effect of viloxazine on [^11^C]CIMBI-36 BP_ND_

Robust dose dependent changes from baseline in BP_ND_ (defined as ratio of bound to unbound [^11^C]CIMBI-36) were seen post-viloxazine administration in most analyzed brain regions (Table 1). Viloxazine displayed greater occupancy (higher % reduction in BP_ND_ [ΔBP_ND_ (%)]) in the choroid plexus (5-HT_2C_ receptor-rich area) than in the cortex (a 5-HT_2A_ receptor-rich area) (Table 1). At the highest dose studied (12 mg/kg), the reduction in BP_ND_ reached approximately 100% in the choroid plexus, while lower occupancy (38% and 42%) was seen in the cortex. In the hippocampus, an area with mixed 5-HT_2A_ and 5-HT_2C_ receptors, reduction was intermediate (58% and 69%). It should be noted that NHP003 unexpectedly displayed notably lower baseline BP_ND_ values compared to other 3 subjects (especially in the cortical regions where this subject’s BP_ND_ was almost 3 SDs lower than the average for the other subjects). Possible reasons for these results are outlined in the limitations section.

**Table 1:** BP_ND_ and ΔBP_ND_ from baseline (%) values of ROIs for each NHP and PET scan for MA1.

NHP	Scan	Viloxazine dose (mg/kg)	Cortical ^a^		Hippocampus ^b^		Choroid plexus	
			BP _ND_	ΔBP _ND_ (%)	BP _ND_	ΔBP _ND_ (%)	BP _ND_	ΔBP _ND_ (%)
**001**	PET1 (BL)		1.68	–	1.76	–	1.18	–
	PET2	12.0	1.04	38%	0.74	58%	0.04	103%
	PET3	6.0	1.14	32%	0.96	45%	0.29	76%
	PET4	4.5	1.45	14%	0.88	51%	0.39	67%
**002** ^**c**^	PET1 (BL)	–	1.55	–	1.61	–	0.82	–
	PET2	12.0	0.90	42%	0.50	69%	0.07	92%
**003**	PET1 (BL)	–	0.98	–	1.47	–	0.66	–
	PET2	3.0	1.08	−10%	0.57	61%	0.18	72%
	PET3	6.0	0.83	15%	1.03	30%	0.12	82%
	PET4	1.0	1.19	−22%	1.03	30%	0.67	−1%
**004**	PET1 (BL)	–	2.08	–	1.49	–	1.13	–
	PET2	3.0	1.56	25%	0.82	45%	0.46	60%
	PET3^d^	1.0	–	–	–	–	–	–

^a^Cortical ROI includes frontal cortex, temporal cortex, limbic structures, parietal cortex, occipital cortex, cingulate cortex, and sensorimotor cortex.

^b^Manually delineated by an analyst using ITKSnap® software.

^c^NHP002 was withdrawn from the study after baseline and a 12.0 mg/kg scan. While preparing the subject for an additional PET scan, they experienced issues with respiration and a problematic intubation which required that the subject be euthanized. The subject was not exposed to viloxazine during this occasion and was replaced by another subject.

^d^Subject 004 PET 3 (S4P3) MA1 V_T_ non-linear fit estimates were not considered to be reliable (coefficient of variation of the fit >50%) and so were not reported.

Abbreviations: NHP = non-human primate subject; BL = baseline; PET = positron emission tomography; BP_ND_ = non-displaceable binding potential; ΔBP_ND_ (%) = percent change in non-displaceable binding potential.

### PK-occupancy analysis of viloxazine at 5-HT_2A_ and 5-HT_2C_ receptor-rich regions

**Figure 4:**
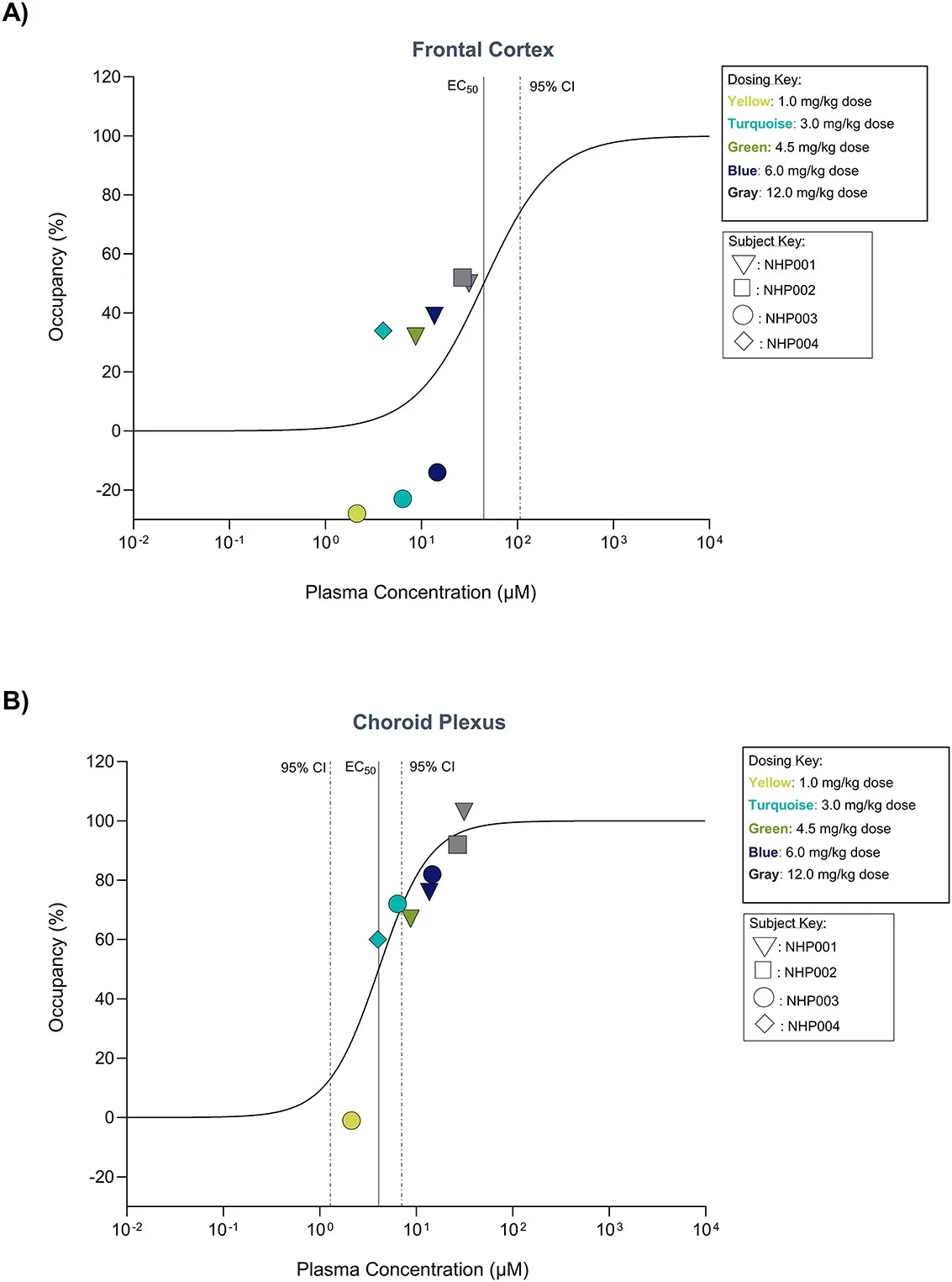
Pharmacokinetic-occupancy analyses in the (A) frontal cortex and (B) choroid plexus. Percentage reduction in BP_ND_ and viloxazine plasma concentration was measured near the start time of the PET scan (40 min after viloxazine infusion and 15 min after [^11^C]CIMBI-36 injection). Occupancy of viloxazine in the frontal cortex and choroid plexus was acquired from 8 PET scans at different unbound plasma concentrations of viloxazine (arising from 1.0, 3.0, 4.5, 6.0, and 12.0 mg/kg doses) for 4 animals (NHP001, 002, 003, and 004). The solid vertical line indicates EC_50_ of viloxazine; the dotted vertical lines indicate 95% CIs. The lower 95% CI in the frontal cortex (A) is less than 10^−2^ and therefore is not present on this graph. Abbreviations: CI, confidence interval; PET = positron emission tomography.

ΔBP_ND_ or target occupancy (%) was plotted against the estimated unbound plasma concentrations of viloxazine obtained near the time of scan start (40 min after viloxazine infusion and 15 min after [^11^C]CIMBI-36 injection) and fitted to a standard occupancy model to obtain EC_50_ of viloxazine in cortical (Figure 4a) and choroid plexus (Figure 4b) regions, representative of occupancy of 5-HT_2A_ and 5-HT_2C_ receptors, respectively. This 40-min plasma timepoint was chosen as a representative timepoint as plasma concentrations stayed fairly constant over time as noted above (Figure 2a). Target occupancy appeared to fit a standard occupancy model for the choroid plexus (5-HT_2C_ receptor-rich area) and to a lesser but acceptable extent for the frontal cortex (5-HT_2A_ receptor-rich area). EC_50_ was estimated to be 45.1 μM (95% confidence interval [CI], −17.7 to 107.9 μM) in the frontal cortex and lower in the choroid plexus (4.1 μM [95% CI, 1.3-7.0 μM]), suggesting that viloxazine has greater occupancy at 5-HT_2C_ relative to 5-HT_2A_ consistent with its in vitro binding profile.

NHP003 demonstrated some negative occupancy values (ie, post dose BP_ND_ was higher for some scans than the baseline BP_ND_). To evaluate the potential contribution of the occupancy values from NHP003 on our estimated EC_50_’s for the frontal cortex and choroid plexus, we recalculated without data from NHP003. While removal of data for NHP003 left-shifted the occupancy (%) vs plasma concentration curves, the EC_50_ and selectivity estimates did not substantially change (Supplemental Figure S12). For example, without NHP003, selectivity of viloxazine remained higher for the choroid plexus (EC_50_ = 3.1 μM, 95% CI, 1.3-5.0 μM) than for the frontal cortex (EC_50_ = 21.7 μM, 95% CI, 8.7-34.6 μM). These EC_50_ estimates were ~25% lower for the choroid plexus and ~50% lower for the frontal cortex but still remained within the 95% CIs of the full dataset and within the same order of magnitude as the overall estimates.

## Discussion

Our study shows that viloxazine reduces the binding of [^11^C]CIMBI-36 at 5-HT_2_ receptors in the brain of cynomolgus monkeys, a species with close physiology to humans, with a 10- to 20-fold higher affinity for 5-HT_2C_ receptors than for 5-HT_2A_ receptors. At unbound viloxazine plasma concentrations comparable with those clinically relevant for the treatment of ADHD, 5-HT_2C_ occupancy levels between 60% and 72% were observed. In the same range of concentrations, viloxazine reduced [^11^C]CIMBI-36 BP_ND_ in the 5-HT_2A_ rich frontal cortex (a brain area relevant to the pathophysiology of ADHD) by 14%-25%. Reduction in [^11^C]CIMBI-36 BP_ND_ may be a combination of a direct occupancy of 5-HT_2A_ receptors by viloxazine and an increase in extracellular 5-HT release. This notion is based on the relatively low affinity of viloxazine for 5-HT_2A_ receptors found in in vitro studies, the evidence from in vivo microdialysis experiments showing that viloxazine increases extracellular 5-HT in this region, and the previously demonstrated sensitivity of [^11^C]CIMBI-36 binding to increases in extracellular 5-HT in the cortical regions.^11^^,^^12^^,^^21^ We discuss this notion in the paragraph below.

Based solely upon the results of this experiment, it might be inferred that viloxazine binds to both 5-HT_2C_ and 5-HT_2A_ receptors.^16^^,^^24^^,^^25^ Although our study showed that viloxazine blocked the binding of [^11^C]CIMBI-36 in all brain regions tested, the highest receptor occupancy and clearest dose response occurred in the choroid plexus, a region reflecting the occupancy of 5-HT_2C_ receptors, with occupancy of the 5-HT_2A_ receptors being less robustly estimated. Our best estimates indicate an approximate 10-fold difference in affinity in vivo between the 2 receptor subtypes. Prior in vitro studies indicated an approximately 25-fold lower affinity for viloxazine at 5-HT_2A_ (K_i_ = 16.0 μM), compared to 5-HT_2C_ (K_i_ = 0.66 μM)^12^^,^^13^ This disparity between in vitro and in vivo affinity ratios for viloxazine at the two 5-HT_2_ receptor subtypes raises the possibility that another mechanism besides direct viloxazine binding may be relevant. A recently published in vivo microdialysis experiment demonstrated statistically significant increases in extracellular 5-HT (as well as NE and DA) in the rat prefrontal cortex at clinically relevant concentrations of viloxazine.^11^ It is possible that augmentation of extracellular 5-HT in 5-HT_2A_ rich regions is due to viloxazine binding to other 5-HT receptors including 5-HT_7_ and 5-HT_2B_ which are known to increase 5-HT in the PFC. Previous preclinical and clinical studies evaluated the sensitivity of [^11^C]CIMBI-36 binding to extracellular 5-HT concentrations using amphetamine and fenfluramine challenges. These studies demonstrated that [^11^C]CIMBI-36 binding was consistently reduced following administration of high doses of 5-HT–releasing agents such as dexamphetamine and fenfluramine.^21^^,^^26–28^ The data from this study cannot provide a definitive conclusion as to the contributions of direct occupancy and increased 5-HT concentration from viloxazine in the cortical regions, and both mechanisms may well contribute to the effects seen.

**Figure 5:**
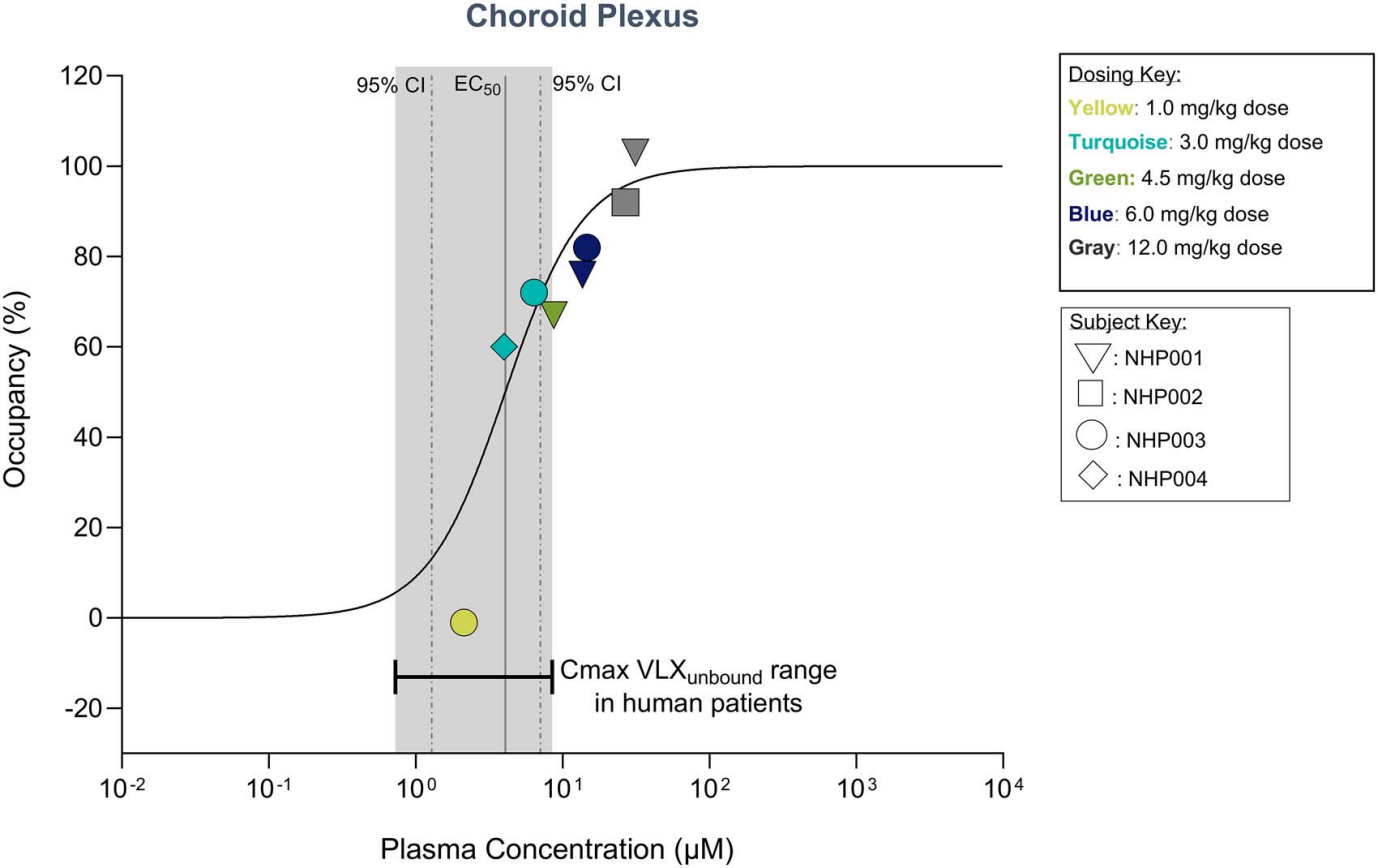
Comparison of NHP plasma concentrations of VLX_unbound_ to the *C*_max_ effective dose range (0.8-7.2 μM) in pediatric and adult patients using approved doses of viloxazine ER for ADHD. Human plasma concentrations of viloxazine were determined from population pharmacokinetic modeling, for approved viloxazine ER doses in children (6-11 years), adolescents (12-17 years), and adults (≥ 18 years) with ADHD. NHP plasma concentrations were determined for all 4 nonhuman subjects at select doses at 1, 3, 4.5, 6, 9, and/or 12 mg/kg of viloxazine, VLX_unbound_ plasma was measured from blood samples taken 40 min after VLX infusion and 15 min after [^11^C]CIMBI-36 injection. Abbreviations: ADHD = attention-deficit/hyperactivity disorder; ER = extended-release; NHP = non-human primates; PET = positron emission tomography; VLX = viloxazine.

The estimated unbound plasma concentrations of viloxazine in NHPs seen in our study (*C*_max_ range: 1.04-31.45 μM) overlapped with unbound plasma concentrations obtained from pediatric and adult population pharmacokinetic models (*C*_max_ 0.8-7.2 μM), that were developed using steady-state plasma concentrations from over 500 participants in phase 3, ADHD clinical trials (Supernus data on file). Despite being calculated from a low sample size resulting in only partial sampling of occupancy curves, the estimated in vivo EC_50_ value in the 5-HT_2C_-rich choroid plexus (4.1 μM [95% CI, 1.3-7.0 μM]) is comparable to that observed from in vitro studies (EC_50_: 1.6 μM)^12^^,^^14^ perhaps suggesting 5-HT_2C_ receptor occupancy at clinically relevant doses (Figure 5). Viloxazine displayed ~50% occupancy of 5-HT_2C,_ within the clinically relevant range indicating that viloxazine could occupy and engage 5-HT_2C_ receptors in humans and therefore may contribute to the clinical efficacy of viloxazine in the treatment of ADHD. This experiment was submitted to the FDA as part of a label change application for viloxazine, which resulted in 5-HT_2C_ partial agonism being added to the existing descriptions of NET inhibition in the pharmacodynamics section of the prescribing information noting relevant pharmacologic effects of the molecule.^29^

### Limitations

The radioligand agonist chosen for this study, [^11^C]CIMBI-36, binds 5-HT_2C_ receptors at the highest levels in the choroid plexus, however, this region of the brain differs structurally from other brain regions, such as the hippocampus, striatum, and midbrain, where we believe viloxazine may have clinically relevant effects on 5-HT_2C_ receptors in the context of ADHD, as well as lying outside the blood–brain barrier. These differences may lead to differences in occupancy between the choroid plexus and other brain regions; however, viloxazine does not appear to be limited in brain access through the blood brain barrier, so the impact of this factor may not be large. This study was conducted in a limited number of animals, with the data therefore displaying moderate variability in the estimates of the EC_50_ at the 2 areas of interest. A limited number of doses were tested in this study resulting in partial sampling of occupancy curves. In determining clinically relevant plasma concentrations, it was assumed that viloxazine distribution between plasma and brain interstitial fluid is similar in humans and NHPs and that the estimated relationship between plasma concentration and brain target occupancy after a single dose is reflective of repeat dosing (ie, that viloxazine is able to diffuse directly between plasma and brain). These assumptions are not unusual in this field, and we have no knowledge of any data that would conflict with these assumptions; however, we have not conducted studies to test these assumptions. In addition, only one baseline scan was conducted per NHP and unusual variability in the estimation of the baseline BP_ND_ may have contributed to the subsequent occupancy estimates. In particular, the low baseline [^11^C]CIMBI-36 BP_ND_ in NHP003 may have reflected experimental variability or baseline instability that could have affected post-dose occupancy estimates in this subject. Nevertheless, analysis of results omitting data from subject NHP003 did not change the conclusions of the study. Finally, we are unable to definitively determine that [^11^C]CIMBI-36 displacement in the cortex is due to viloxazine-induced increases in endogenous 5-HT and not direct viloxazine occupancy of 5-HT_2A_. We hypothesized that the mechanism is indirect (ie, increased 5-HT concentration) based on previous findings from in vitro binding affinity and in vivo microdialysis experiments showing that viloxazine has low binding affinity for 5-HT_2A_, but is able to increase 5-HT levels in the prefrontal cortex of rats.

### Summary and future directions

Overall, this study shows that, at concentrations clinically relevant for the treatment of ADHD, viloxazine binds to 5-HT_2C_ receptors in the NHP brain. Due to low binding affinity of viloxazine for 5-HT_2A_, it is possible that viloxazine-induced displacement of [^11^C]CIMBI-36 at 5-HT_2A_ receptors in the frontal cortex is due to viloxazine-induced increases in extracellular 5-HT, which may be secondary to viloxazine action at other 5-HT receptors (ie, 5-HT_2B_ and 5-HT_7_). These effects may contribute to the clinical efficacy of viloxazine in the treatment of ADHD as 5-HT_2C_ agonists, such as lorcaserin, have shown a reduction in impulsive behaviors associated with overeating and addiction and 5-HT_2C_ antagonism has been shown to increase impulsivity in animal models.^30–33^ In addition, extracellular increases in 5-HT in the frontal cortex may also play a role in clinical efficacy as this region is key in ADHD pathology. These results imply that the classification of viloxazine as a “selective NRI” is insufficient. Viloxazine may be better described as a “NET inhibitor” (since NET inhibition increases synaptic DA in addition to NE) with targeted 5-HT receptor effects, which include partial agonism of 5-HT_2C_. In agreement with this description, viloxazine has recently been classified as norepinephrine, serotonin multimodal agent by the Neuroscience Based Nomenclature system with its mode of action listed as a NRI and 5-HT_2C_ partial agonist. Additional in vivo studies are needed to determine whether viloxazine directly occupies 5-HT_2A_ receptors or whether displacement of [^11^C]CIMBI-36 is due to increases in endogenous 5-HT due to clinically relevant occupancy of other receptors such as 5-HT_2B_ and/or 5-HT_7_ (as suggested by in vitro binding experiments). Further exploration is also needed to determine the contributions of 5-HT receptor activities to the treatment of ADHD and its associated symptoms.

## Supplementary Material

VLX_PET_Manuscript_IJNP_Supplementary_Materials_Final_pyag034

## Data Availability

The data underlying this article will be shared on reasonable request to the corresponding author.
