# Supplementary material for: Viloxazine occupies the 5-HT2C receptor in the Macaca fascicularis brain in vivo: a [11C]CIMBI-36 positron emission tomography study
Source: Int J Neuropsychopharmacol. 2026 Jun 23;29(8):pyag034. doi: 10.1093/ijnp/pyag034 (PMC13499530; doi:10.1093/ijnp/pyag034)
Supplement: Supplementary file 1 [file vlx_pet_manuscript_ijnp_supplementary_materials_final_pyag034.docx]

**Supplemental Methods:**

*Animals*

The monkeys were housed in groups of up to six individuals in a space of about 12 – 20 m^2^ for combined indoor / outdoor living (at least 2 m^2^ per NHP). The cage height was 2.5 – 3.7 m. The animals’ environment was structurally enriched to satisfy the animals' physiological and behavioral needs. Animals were provided a diet of 50% nutritionally balanced pellets (morning), the rest were fresh vegetables (afternoon). Animals had access to water ad libitum.

NHP002 was withdrawn from the study due to an issue unrelated to the test articles. While preparing the subject for a PET scan, the subject experienced issues with respiration after problematic intubation, which required that the subject be euthanized. The subject was not exposed to viloxazine during this occasion and was replaced by another subject.

*Preparation of [^11^C]CIMBI-36*

[^11^C]CIMBI-36 was radiolabeled using [^11^C]methyl triflate which was prepared from [^11^C]methane by converting [^11^C]methane into [^11^C]methyl iodide by high temperature iodination in a graphase recirculation system and then transforming it into [^11^C]methyl triflate. Briefly, [^11^C]CH_3_OTf was trapped in a reaction vessel at room temperature (RT) with the precursor CIMBI-37 (0.4–0.6 mg), acetonitrile (300 μL), acetone (300 μL) and aqueous sodium hydroxide (3 M) (2–3 μL). After the reaction (ca. 1.5 min), 0.6 mL of trifluoroacetic acid/acetonitrile (1/2 v/v mixture) was added to the reaction vessel and the mixture was heated to 80 °C for 5 min. After cooling to RT, the reaction mixture was neutralized with aqueous sodium hydroxide (3 M) (1500 μL) and purified using a HPLC system. The desired fraction was collected into a vial containing 60 mL of water. The resulting solution was pushed through tC18 Short SPE cartridge (Waters), and the cartridge was rinsed with 8 mL of sterile water. The product was then eluted with 3.0 mL of 30% of ethanol in propylene glycol and collected in a sterile vial pre-filled with 10 mL sterile physiological phosphate buffer or saline. The solution was filtered through a Millex-GV filter into the product vial. The resulting product was analysed for radiochemical purity and specific radioactivity. Radiochemical purity was in the range of 90 to 99%, and molar activity was in the range of 191 to 1105 GBq/μmol at the time-of-injection, corresponding to the injected mass range of 0.36 to 0.04 μg.

*Preparation of viloxazine hydrochloride*

Viloxazine hydrochloride was formulated in a standard volume of sterile saline based on weight of the NHP to the appropriate concentration (1, 3, 4.5, 6, or 12 mg/kg dose) on the day of scans and filtered using a Millex-GV sterile filter. Concentrations were verified before and after sterile filtration. Analysis was done using a HPLC system equipped with a XBridge C18 Shield 50 x 10 mm. Acetonitrile 0.05 M aqueous phosphoric acid (18:82) was used as mobile phase with flow set to 5 mL/min for XBridge UV detector, which was set to 254 nm. Pre-treatment preparation was deemed acceptable if deviation of calculated concentration vs standard solution was <10% and loss on sterile filtration did not exceed 10%.

*Blood sampling for determination of [11C]CIMBI-36 radioactivity and radiometabolite analysis*

To continuously collect whole blood, a cannula was inserted in the external iliac or femoral artery and an automatic blood-sampling system was used for the first 3 minutes after radioligand injection. Discrete blood samples were also manually drawn at 5, 15, 30, 45, 60, 75, 90 and 120 min after injection of [^11^C]CIMBI-36. Protein precipitation sample preparation was coupled with reversed-phase radio-HPLC in order to quantitate [^11^C]CIMBI-36 and its radioactive metabolites in the blood samples. Plasma was separated from whole blood samples using centrifugation, samples were mixed with 1.4x volume of acetonitrile, and again centrifuged (2000 rpm × 4 min). The supernatants were diluted with water and injected into a reversed phased radio-HPLC. Chromatographic separation was obtained using an ACE C18 column (250 mm x 10 mm I.D). A gradient elution of acetonitrile (A) and ammonium formate (AMF) 0.1 M were used as the mobile phase at 5.0 mL/min, with the following gradient; 0–4.0 min, (A/AMF) 60:40 to 90:10 v/v; 4.0–6.0 min, (A/AMF) 90:10 v/v for [^11^C]CIMBI-36 and 0–4.0 min, (A/AMF) 65:35 to 90:10 v/v; with an Agilent binary pump (Agilent 1200 series) coupled to a manual injection valve (7725i, Rheodyne), 5.0 mL loop and a radiation detector (Oyokoken, S-2493Z) housed in a shield of 50 mm thick lead measurements with 10 second radio-detector accumulation. In addition, a ChemStation Rev. B.04.03 (Agilent) was used to aid collecting and controlling of the data. Peaks for radioactive compounds eluting from the column were integrated and their areas were expressed as a percentage of the sum of the areas of all detected radioactive compounds, with consideration of a time decay correction.

*Determination of viloxazine plasma concentrations*

Viloxazine was extracted from plasma by protein precipitation using 95% acetonitrile and 5% dimethyl sulfoxide (DMSO). After vortexing and centrifugation, the plasma supernatant was injected onto a Chiral-AGP 150 x 4.0 mm, 5 μm analytical column. Viloxazine analytes were eluted from the chromatographic column in an aqueous and acetonitrile mobile phase and were detected with MS/MS in positive ion mode. The fraction of unbound viloxazine in cynomolgus plasma was determined using an in vitro protein binding assay using, more specifically an equilibrium dialysis assay following all standard operating procedures of Wuxi AppTec (New Jersey, USA). Viloxazine (2 µM) was spiked into plasma and dialyzed against an equal volume of phosphate buffer saline at ph 7.4 in a HTDialysis device (12-14 kDa cut-off) for 2, 4 and 6 h in a humidified incubator (5% CO_2_) at 37^o^C. Concentrations of viloxazine in both compartments (donor and acceptor) were determined by a liquid chromatography-tandem triple quadrupole mass spectrometry (LC-MS/MS) method. The percentage of unbound viloxazine in cynomolgus plasma was estimated to be 57.3 %. The total unbound concentration of viloxazine was estimated by adjusting the total plasma concentrations of viloxazine by the fraction of unbound viloxazine in NHP plasma (f_u,plasma_=0.57)

**Supplemental Tables:**

Supplemental Table 1: Animal details

| Animal ID | Sex | Age | Weight (kg) |
| --- | --- | --- | --- |
| NHP001 | Male | 12 | 8.7 |
| NHP002 | Female | 11 | 6.4 |
| NHP003 | Male | 13 | 9.2 |
| NHP004 | Female | 11 | 5.9 |

Supplemental Table 2: Total volume of distribution (V_T_ units mL/cm^3^) derived from Multilinear Analysis 1 (MA1) after the injection of [^11^C]CIMBI-36 in NHPs and pretreatment with viloxazine, for each NHP and PET scan.

| **NHP** | **Scan** | **Viloxazine**  **dose (mg/kg)** | **Cortical^a^** | **Hippocampus^b^** | **Choroid plexus** | **Viloxazine plasma conc. (ng/mL)** |
| --- | --- | --- | --- | --- | --- | --- |
| 001 | PET1 (BL) | - | 11.56 | 11.92 | 9.4 | - |
|  | PET2 | 12.0 | 15.03 | 12.84 | 7.06 | 13050 |
|  | PET3 | 6.0 | 9.46 | 8.7 | 5.69 | 5670 |
|  | PET4 | 4.5 | 15.26 | 11.57 | 8.64 | 3620 |
| 002^c^ | PET1 (BL) | - | 13.56 | 13.9 | 9.68 | - |
|  | PET2 | 12.0 | 14.43 | 11.4 | 8.13 | 11160 |
| 003 | PET1 (BL) | - | 8.07 | 10.08 | 6.78 | - |
|  | PET2 | 3.0 | 6.49 | 4.91 | 3.69 | 2660 |
|  | PET3 | 6.0 | 6.71 | 7.46 | 4.11 | 6080 |
|  | PET4 | 1.0 | 8.31 | 7.7 | 6.34 | 886 |
| 004 | PET1 (BL) | - | 17.8 | 14.38 | 12.32 | - |
|  | PET2 | 3.0 | 15.82 | 11.28 | 9.01 | 1648 |
|  | PET3^d^ | 1.0 | 54.75 | 27.84 | 17.97 | 434 |

Viloxazine plasma concentration (ng/mL) from sample obtained at 15 minutes post start of scanning.

^a^ Cortical ROI includes frontal cortex, temporal cortex, limbic structures, parietal cortex, occipital cortex, cingulate cortex, and sensorimotor cortex.

^b^ Manually derived.

^c^ NHP002 was withdrawn from the study after baseline and a 12.0 mg/kg scan. While preparing the subject for an additional PET scan, they experienced issues with respiration and a problematic intubation which required that the subject be euthanized. The subject was not exposed to viloxazine during this occasion and was replaced by another subject

^d^ Subject 004 PET 3 (S4P3) MA1 V_T_ estimates are not well determined (e.g., high SD) and outside the plausible range.

NHP: Non-human primate subject; BL: Baseline; PET: Positron Emission Tomography; Conc: concentration

Supplemental Table 3: NHP scan and injection record.

| ID # | Scan # | Date | Drug | Dose (mg/kg) | Ligand | Activity (Mbq/mCi) |
| --- | --- | --- | --- | --- | --- | --- |
| 001 | PET1^a^ | 27JAN2022 | none | (baseline scan) | [^11^C]CIMBI-36 | 141/3.81 |
| 001 | PET2^a^ | 27JAN2022 | viloxazine | 12.0 | [^11^C]CIMBI-36 | 141/3.81 |
| 002^b^ | PET1^a^ | 31JAN2022 | none | (baseline scan) | [^11^C]CIMBI-36 | 145/3.92 |
| 002^b^ | PET2^a^ | 31JAN2022 | viloxazine | 12.0 | [^11^C]CIMBI-36 | 142/3.84 |
| 003 | PET1^a^ | 11MAR2022 | none | (baseline scan) | [^11^C]CIMBI-36 | 112/3.03 |
| 003 | PET2 | 11MAR2022 | viloxazine | 3.0 | [^11^C]CIMBI-36 | 121/3.27 |
| 001 | PET3 | 22APR2022 | viloxazine | 6.0 | [^11^C]CIMBI-36 | 171/4.62 |
| 003 | PET3 | 25APR2022 | viloxazine | 6.0 | [^11^C]CIMBI-36 | 200/5.40 |
| 004 | PET1 | 13MAY2022 | none | (baseline scan) | [^11^C]CIMBI-36 | 180/4.86 |
| 004 | PET2 | 13MAY2022 | viloxazine | 3.0 | [^11^C]CIMBI-36 | 181/4.89 |
| 003 | PET4 | 10JUN2022 | viloxazine | 1.0 | [^11^C]CIMBI-36 | 192/5.19 |
| 001 | PET4 | 15AUG2022 | viloxazine | 4.5 | [^11^C]CIMBI-36 | 172/4.65 |
| 004 | PET3 | 22AUG2022 | viloxazine | 1.0 | [^11^C]CIMBI-36 | 194/5.24 |

^a^Due to technical issues, metabolite analysis was performed only up to 30 min post injection for PET1 and PET2 in Subject 001 and for PET1 in Subject 002. For PET1 in Subject 003, samples at 90 min were too noisy to be reliable.

^b^NHP002 was withdrawn from the study after baseline and a 12.0 mg/kg scan. While preparing the subject for an additional PET scan, the subject experienced respiratory difficulties after a problematic intubation, which required that the subject be euthanized. The subject was not exposed to viloxazine during this occasion.

**Supplemental Figures:**

Supplemental Figure 1: Generated blood input functions for NHP001. A) Parent fraction, B) parent plasma input, and C) plasma over blood.

Supplemental Figure 2: Generated blood input functions for NHP002. A) Parent fraction, B) parent plasma input, and C) plasma over blood.

Supplemental Figure 3: Generated blood input functions for NHP003. A) Parent fraction, B) parent plasma input, and C) plasma over blood.

Supplemental Figure 4: Generated blood input functions for NHP004. A) Parent fraction, B) parent plasma input, and C) plasma over blood.

Supplemental Figure 5: [^11^C]CIMBI-36 PET images of NHP002 showing expected radioligand uptake at **A)** baseline, and after administration of **B)** 12 mg/kg of viloxazine scaled to standardized uptake values (SUVs) and superimposed on a MRI template of the brain.

Supplemental Figure 6: [^11^C]CIMBI-36 PET images of NHP003 showing expected radioligand uptake at **A)** baseline, and after administration of **B)** 1 mg/kg, **C)** 3 mg/kg, and **D)** 6 mg/kg of viloxazine scaled to standardized uptake values (SUVs) and superimposed on a MRI template of the brain.

Supplemental Figure 7: [^11^C]CIMBI-36 PET images of NHP004 showing expected radioligand uptake at **A)** baseline, and after administration of **B)** 1 mg/kg and **C)** 3 mg/kg of viloxazine scaled to standardized uptake values (SUVs) and superimposed on a MRI template of the brain.

Supplemental Figure 8: Representative [^11^C]CIMBI-36 time-activity curves (TACs) with acceptable MA1 model fits at the **A)** frontal cortex, **B)** choroid plexus, and **C)** hippocampus in comparison to the cerebellum used as a reference region at baseline and after administration of 4.5, 6, and 12 mg/kg of viloxazine in representative subject, NHP001.

Supplemental Figure 9: Representative [^11^C]CIMBI-36 time-activity curves (TACs) with acceptable MA1 model fits at the **A)** frontal cortex, **B)** choroid plexus, and **C)** hippocampus in comparison to the cerebellum used as a reference region at baseline and after administration of 12 mg/kg of viloxazine in representative subject, NHP002.

Supplemental Figure 10: Representative [^11^C]CIMBI-36 time-activity curves (TACs) with acceptable MA1 model fits at the **A)** frontal cortex, **B)** choroid plexus, and **C)** hippocampus in comparison to the cerebellum used as a reference region at baseline and after administration of 1, 3, and 6 mg/kg of viloxazine in representative subject, NHP003.

Supplemental Figure 11: Representative [^11^C]CIMBI-36 time-activity curves (TACs) with acceptable MA1 model fits at the **A)** frontal cortex, **B)** choroid plexus, and **C)** hippocampus in comparison to the cerebellum used as a reference region at baseline and after administration of 1 and 3 mg/kg of viloxazine in representative subject, NHP004.

**A)**

**B)**

Supplemental Figure 12: Comparison of pharmacokinetic-occupancy analyses for A) frontal cortex and B) choroid plexus with and without NHP003. Percentage reduction in BP_ND_ % and viloxazine plasma concentration was measured near the start time of the PET scan (40 minutes after viloxazine infusion and 15 minutes after [^11^C]CIMBI-36 injection). Occupancy of viloxazine in the frontal cortex and choroid plexus was acquired from 8 PET scans at different unbound plasma concentrations of viloxazine (arising from 1.0, 3.0, 4.5, 6.0, and 12.0 mg/kg doses) for 4 animals (NHP001, 002, 003, and 004). The black curves represent all data points incorporated, while the red curves represent occupancy with NHP003 data points removed. The solid vertical line indicates EC_50_ of viloxazine; the dotted vertical lines indicate 95% confidence intervals (CIs) (black represents all data points, red represents data points from NHP003 removed). The lower 95% CI in the frontal cortex (A) with all data points is less than 10^-2^ and therefore is not present on this graph.
